# Sutureless Aortic Prosthesis Valves versus Transcatheter Aortic Valve Implantation in Intermediate Risk Patients with Severe Aortic Stenosis: A Literature Review

**DOI:** 10.3390/jcm13185592

**Published:** 2024-09-20

**Authors:** Laura Asta, Adriana Sbrigata, Calogera Pisano

**Affiliations:** 1 Department of Cardiac Surgery, Clinical Mediterranean, 80122 Naples, Italy; astalaura92@gmail.com; 2 Cardiac Surgery Unit, Department of Precision Medicine in Medical Surgical and Critical Area (Me.Pre.C.C.), University of Palermo, 90134 Palermo, Italy; adriana.sbrigata@gmail.com

**Keywords:** aortic stenosis, transcatheter aortic valve implantation, sutureless aortic prosthesis

## Abstract

Aortic stenosis remains the most frequently occurring valvular pathology in the elderly population of Western countries. According to the latest guidelines, the therapeutic choice of aortic stenosis depends on the age of the patient (<75 years or >75 years) and the risk class (STS-Prom/Euroscore II < o >4%). Therefore, if the surgical indication is clear in young and low-risk patients and percutaneous treatment is the gold standard in older and high-risk patients, the therapeutic choice is still debated in intermediate-risk patients. In this group of patients, aortic valve stenosis treatment depends on the patient’s global evaluation, the experience of the center, and, no less importantly, the patient’s will. Two main therapeutic options are debated: surgical aortic valve replacement with sutureless prosthesis versus transcatheter aortic valve implantation. In addition, the progressive development of mininvasive techniques for aortic valve surgery (right-anterior minithoracotomy) has also reduced the peri- and post-operative risk in this group of patients. The purpose of this review is to compare sutureless aortic valve replacement (SuAVR) versus TAVI in intermediate-risk patients with severe aortic stenosis.

## 1. Introduction

The most widespread valvulopathy in the Western world is the stenosis of the aortic valve (AV), with a prevalence of 3% for individuals over the age of 75 years [1]. The incidence is growing as a reflection of the rapid aging of the population [2]. Surgical aortic valve replacement (SAVR) has always been the most beneficial therapeutic option in terms of quality of life and clinical outcomes in symptomatic patients [3]. However, in the last two decades, the treatment of aortic valve pathology has been constantly evolving with an increase in the use of transcatheter aortic valve replacement (TAVR) not only in high-risk patients but also in intermediate- and low-risk patients [4]. As TAVR has become more established, newer surgical prostheses have been developed with a particular implantation system that does not require sutures [5]. This new aortic prosthesis is called the “sutureless prosthesis”. This prosthesis significantly reduces operative and ischemic times and may improve clinical outcomes, particularly in intermediate-risk patients [6,7,8]. On the other hand, with its compact design and easy handling, sutureless prosthesis facilitates a minimally invasive approach, which has been shown to be of comparable quality to a full sternotomy for AVR [9,10,11,12]. If the surgical indication is clear in young and low-risk patients and percutaneous treatment is the gold standard in older and high-risk patients, the therapeutic choice is still debated in intermediate-risk patients. In this group of patients, aortic valve stenosis treatment is related to the patient’s global evaluation (evaluation in Heart Team), the experience of the center, and, no less importantly, the patient’s will. The purpose of this review is to compare sutureless aortic valve replacement (SuAVR) versus TAVI in intermediate-risk patients with severe aortic stenosis. It is good to specify from the beginning that there are no randomized controlled studies in this somewhat limited group of patients; furthermore, there are relatively few data on SuAVR. On the other hand, in many trials comparing SAVR versus TAVI, sutureless prosthesis valves were excluded.

## 2. Material and Methods

### 2.1. Types and Characteristics of Prostheses

The term sutureless refers to two different valve types: the sutureless Perceval prosthesis (Corcym, London, UK) and the rapid deployment INTUITY Elite prosthesis (Edwards Lifesciences, Irvine, CA, USA). 

**Figure 1:**
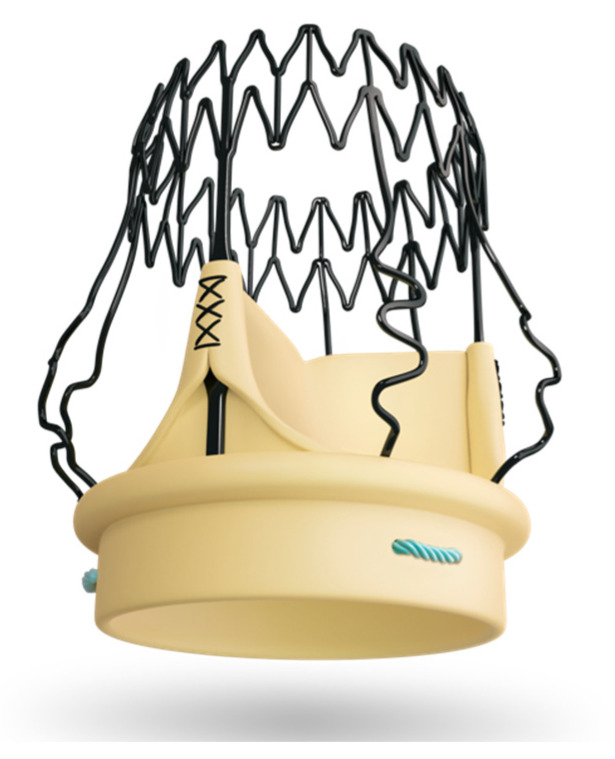
Perceval prosthesis (Corcym, London, UK).

**Figure 2:**
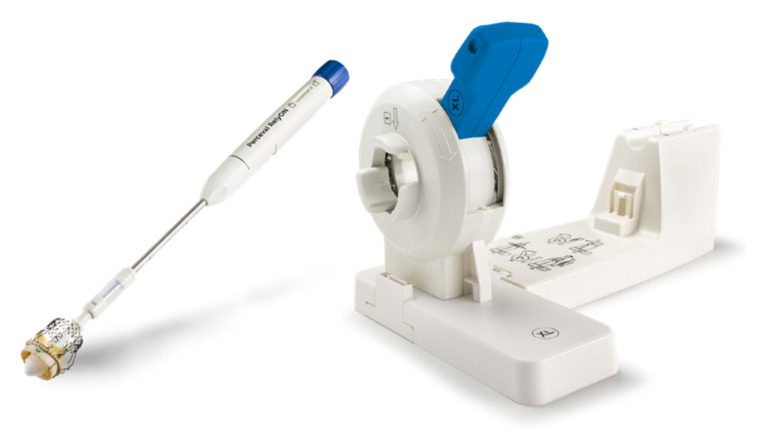
Device used for prosthesis collapse (Corcym, London, UK).

The Perceval prosthesis has a nitinol frame, which guarantees shape memory and superelasticity, and bovine pericardium flaps (Figure 1). The implantation of the prosthesis involves, after the traditional steps (preparation of the surgical access (sternotomy, ministernotomy, right anterior minithoracotomy), cannulation, aortotomy, removal of the native valve, decalcification of the annulus, and accurate sizing), the collapse of the valve by means of an appropriate device that guarantees an atraumatic closure of the prosthesis (Figure 2) and subsequently the positioning of guiding sutures 2–3 mm under leaflet point that guarantee the correct orientation of the prosthesis. Finally, the guiding sutures were removed after releasing the prosthesis from the holder, and a balloon was expanded into the valve at 4 atm for 30 s [13].

**Figure 3:**
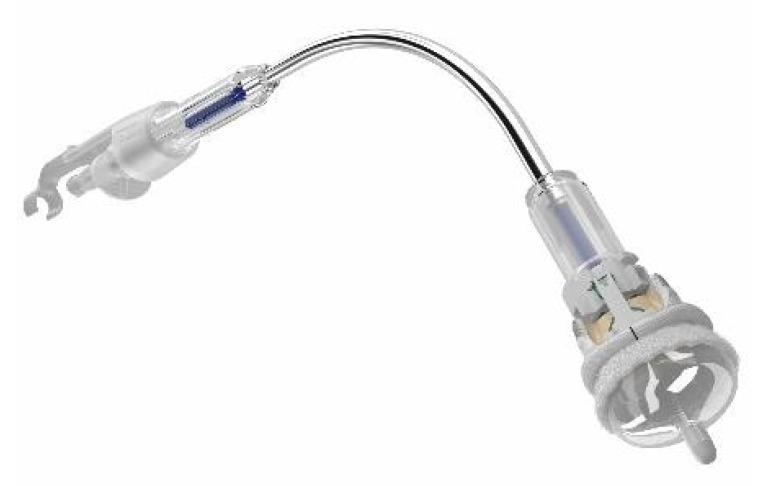
The INTUITY Elite prosthesis (Edwards Lifesciences, Irvine, CA, USA).

The INTUITY Elite (Edwards Lifesciences, Irvine, CA, USA) prosthesis consists of a bovine pericardium valve (Carpentier-Edwards PERIMOUNT type) positioned inside a stent-based balloon-expandable fixation system (Figure 3). After adequate sizing of the prosthesis, three equidistant non-pledged guide sutures are positioned inside the native annulus, corresponding to the markers on the sewing cuff. Subsequently, the INTUITY Elite valve can be parachuted into the native annulus, using the fixation system, advancing the balloon catheter until the “click” of correct positioning. Finally, the balloon is inflated (present inside the same fixation system), and as soon as it is deflated, the balloon is deflated, and the fixation system is removed. The three sutures are tied and cut near the knots [14].

**Figure 4:**
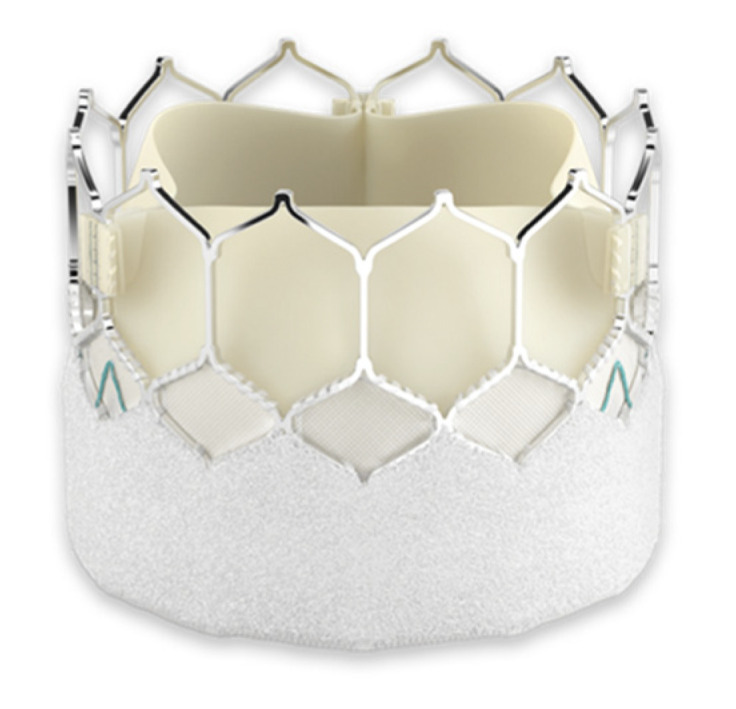
SAPIEN 3 Ultra valve (Edwards Lifesciences Corporation, Irvine, CA, USA).

TAVR uses two main categories of prostheses: balloon-expandable and self-expandable. The third generation of balloon-expandable SAPIEN ^TM^ valves (Edwards Lifesciences Corporation, Irvine, CA, USA) includes the SAPIEN 3 and the SAPIEN 3 Ultra valves, which consist of three bovine pericardial flaps positioned inside of a cobalt–chromium stent. The SAPIEN 3 Ultra prosthesis has an outer skirt that is ~40% taller than the SAPIEN 3, textured polyethylene terephthalate (PET), and a short frame height and open cell geometry to facilitate coronary access (Figure 4).

**Figure 5:**
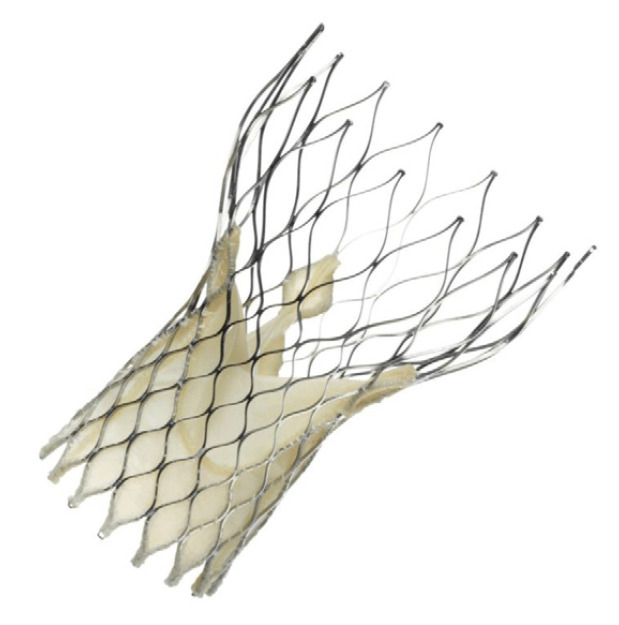
Core Valve^TM^ (Medtronic, Inc., Minneapolis, MN, USA).

Among the self-expanding prostheses, the most widely used is the CoreValve^TM^ (Medtronic, Inc., Minneapolis, MN, USA), which consists of three flaps of porcine pericardium sutured inside a self-expanding, multi-level, radiopaque frame made of Nitinol (Figure 5). The next generation, Evolut Pro, features an external casing that enhances surface contact between the valve and the native aortic annulus to further improve the valve’s sealing performance and allows for recapture and repositioning after deployment [15].

The different structural characteristics of the prostheses allow the diversification of their use based on the anatomical conditions of the patient. Although sutureless prostheses and TAVR share some common conceptual and design aspects (self- or balloon-expandable and the presence of a stent with a biological prosthesis inside), the implantation technique already differentiates their use. The need for a surgical approach (potentially with minimally invasive techniques, such as ministernotomy or right anterior minithoracotomy) means that sutureless prostheses are not recommended for a category of patients with high surgical risk, just as non-viable peripheral vascular accesses are a contraindication to TAVR. In choosing the strategy, some anatomical characteristics of the aortic root must be evaluated:

- The size of the ring (in case of an annulus that is too small, which would require enlargement procedures, or too large for which size XL prostheses should be used, surgery with a traditional prosthesis is preferable).
- Calcifications (the absence of calcifications can cause a dislocation of the TAVR due to lack of anchoring). Furthermore, failure to remove the calcified native valve increases the risk of stroke as well as being the main cause of PVL and pacemaker implantation in TAVR. In fact, the removal of the valve and accurate decalcification of the annulus (in addition to the possible removal of infected material) allow for better positioning of the prosthesis, as well as ensuring greater durability over time.
- The wall quality (the fragility of the wall suggests the use of a sutureless prosthesis to avoid the risk of rupture or dissection post-TAVR).
- Anatomical anomalies (bicuspid aortic valve or aneurysmal dilations) orient toward the choice of traditional prostheses.
- Porcelain aorta (absolute contraindication to the surgical procedure), while in the presence of calcifications at the level of the aortic root (previous homograft), a sutureless prosthesis is advisable.
- The height of the coronary ostia (a reduced distance between the valve plane and the ostia is a significant risk in the use of stented prostheses such as TAVR or sutureless; therefore, the use of traditional prostheses is preferable).
- The risk of atrioventricular conduction block (due to previous conduction disorders or the presence of calcifications at the level of the interventricular septum) makes surgical treatment with a traditional prosthesis advisable.

Furthermore, in cases where there is an indication for other cardiac surgery procedures (e.g., coronary bypass grafts), it is preferable to use sutureless prostheses to further reduce operating times. Conversely, in the presence of patent coronary bypass grafts, the TAVR approach is preferable [16].

A condition that deserves a separate discussion is in valve-in-valve (Viv) procedures. Although the gold standard in the treatment of prosthetic dysfunction is reoperation, in high-risk patients, ViV-TAVR has increasingly become popular. However, in addition to some conditions that contraindicate this procedure, such as in the presence of a very small annulus, it has emerged that redo-AVR has a higher 30-day mortality rate than ViV-TAVR but comparable if mortality in the entire follow-up is taken into account, in the face of a lower rate of paravalvular leaks, pacemaker implantation, severe patient–prosthesis mismatch and mean post-operative gradient. However, in the therapeutic choice in these cases, the global evaluation of the patient’s clinical and anatomical conditions, as well as the experience of the center, are of absolute importance [17].

### 2.2. Data Sources and Search Strategy

The current literature investigating severe aortic stenosis, intermediate-surgical-risk patients, surgical treatments, transcatheter aortic valve replacement, and minimally invasive aortic valve replacement with sutureless aortic valves was analyzed and contextualized in this review. Specifically, research was conducted on Medline (Pubmed) and Scopus. To review recent studies, we selected scientific papers published in English in the last ten years. We used the following search terms: severe aortic stenosis, transcatheter aortic valve replacement, minimally invasive surgery, sutureless aortic valve prostheses, and cardiac surgery. 

### 2.3. Study Selection

#### 2.3.1. Inclusion Criteria

The inclusion criteria for the included studies in this review were as follows: (1) management of severe aortic stenosis in intermediate-surgical-risk patients; (2) comparison between transcatheter aortic valve implantation and sutureless aortic prosthesis valve in intermediate-surgical-risk patients; and (3) role of minimally invasive surgery to improve clinical outcomes in patients undergoing aortic valve replacement with sutureless aortic valve prostheses.

#### 2.3.2. Exclusion Criteria

Editorials, case reports, letters to the editor, and conference abstracts were excluded from this review.

## 3. Management of Severe Aortic Stenosis According to the International Guidelines

The 2021 ESC/EACTS guidelines [18] for the management of valvular heart diseases advocated that aortic valve interventions must be performed in heart valve centers that declare their local expertise and outcomes data, have active interventional cardiology and cardiac surgical programs on site, and a structured collaborative Heart Team approach (IC); the choice between surgical and transcatheter intervention must be based upon careful evaluation of clinical, anatomical, and procedural factors by the Heart Team, weighing the risks and benefits of each approach for an individual patient (IC); the Heart Team recommendation should be discussed with the patient who can then make an informed treatment choice (IC); SAVR is recommended in younger patients who are low risk for surgery (<75 years and STS-PROM/EuroSCORE II < 4%) or in patients who are operable and unsuitable for transfemoral TAVR [19]; TAVR is recommended in older patients (≥75 years), or in those who are high risk (STS- PROM/EuroSCORE II > 8%) or unsuitable for surgery [20,21,22,23,24,25,26,27,28,29,30]; SAVR or TAVR are recommended for remaining patients according to individual clinical, anatomical, and procedural characteristics [31,32,33,34]. The 2020 ACC/AHA guidelines for the management of patients with heart valve diseases [35] state the following: for symptomatic and asymptomatic patients with severe AS and any indication for AVR who are <65 years of age or have a life expectancy >20 years, SAVR is recommended [36,37,38]; for symptomatic patients with severe AS who are 65 to 80 years of age and have no anatomic contraindication to transfemoral TAVR, either SAVR or transfemoral TAVI is recommended after shared decision-making about the balance between expected patient longevity and valve durability [37,38,39,40,41]; for symptomatic patients with severe AS who are >80 years of age or for younger patients with a life expectancy <10 years and no anatomic contraindication to transfemoral TAVR, transfemoral TAVI is recommended in preference to SAVR [42,43,44,45]. Both guidelines have highlighted age (a surrogate for life expectancy) as the main consideration after accounting for patient preferences, comorbidities, and anatomical characteristics. The age disparities between guidelines and the perceived crudeness of this approach have incited some controversy [46]. The reason for these age disparities is related to the different studies on which the ACC/AHA and the ESC/AHA guidelines are based. The ACC/AHA cites a mix of systematic reviews, RCTs, and observational studies to recommend SAVR for <65 years based on a lack of TAVI follow-up data beyond 5 years, while the ESC/EACTS guidelines cite observational registries that demonstrated a 5-year rate of severe structural valve deterioration of 2.5% and moderate deterioration of 13.3% after TAVR. None of the aforementioned data provide robust evidence for the age thresholds established by the ACC/AHA and ESC/EACTS guidelines. On the contrary, results from the NOTION [47] and UK TAVI trials [48] provided more clarity about low-to-intermediate-risk patients ≥70 years of age with a EuroSCORE II < 4%. Finally, the 5-year results of the SURTAVI [49] trial also support the durability of TAVR in intermediate-risk patients. These findings, in addition to the 5-year UK TAVI and 10-year NOTION results expected shortly, may provide greater clarity to guidelines and the current uncertainty regarding the optimal age threshold for TAVI and the degree to which age should play a factor in the overall decision-making process after considering patient comorbidities, preferences, and anatomical presentation. Accordingly, in our opinion, the choice of the aortic valve treatment (SAVR versus TAVR) should be based on the risk score classification rather than on the age threshold. However, the guidelines do not currently provide clear indications on the use of sutureless or standard prostheses. To date, the only available trial comparing the results of sutureless and standard prostheses is the PERSIST-AVR (Perceval Sutureless Implant Versus Standard-Aortic Valve Replacement), a multicenter randomized control trial that aims to demonstrate the non-inferiority of sutureless prostheses compared to standard prostheses, in the face of shorter operating times [6]. The results that emerged at one year of follow-up show comparable hemodynamic performance (peak and mean gradients, paravalvular and central leaks) between the two different types of prosthesis [50]. Furthermore, the implantation of permanent pacemakers has been seen to be associated with the rate of pre-operative conduction alterations and the size of the XL valve [51]. However, this study includes patients at low surgical risk, and it is therefore necessary to underline how the scientific evidence in intermediate-risk patients is even lower.

## 4. State of the Art in the Management of Intermediate-Surgical-Risk Patients with Severe Aortic Stenosis

If the surgical indication is clear in young and low-risk patients and percutaneous treatment is the gold standard in older and high-risk patients, the therapeutic choice is still debated in intermediate-risk patients. In this group of patients, AV stenosis treatment depends on the patient’s global evaluation, the experience of the center, and, no less importantly, the patient’s will. Two main therapeutic options are debated: surgical aortic valve replacement (SAVR) with sutureless prosthesis versus transcatheter aortic valve implantation (TAVR). The introduction of the new guidelines certainly expands the category of patients subjected to TAVR [18,19,20,21,22,23,24,25,26,27,28,29,30,31,32,33,34,35]. However, the evaluation of the hemodynamic aspects, the durability of the prosthesis, and the rate of complications (mortality and major cerebrovascular events) still remain to be clarified. On the other hand, the “sutureless prosthesis”, by avoiding placement and tying of annular sutures, significantly reduces operative and, more importantly, ischemic times and may improve outcomes [7,8]. Furthermore, sutureless aortic prostheses should be considered in order to minimize operative times and improve outcomes in high-risk patients for whom a long bypass run would be detrimental and in those undergoing complex combined procedures. Finally, with its compact design and easy handling, the sutureless prosthesis facilitates a minimally invasive approach, which has been shown to be of comparable quality to a full sternotomy for AVR [52,53]. In addition to the better results compared to traditional surgery, minimally invasive treatment of the aortic valve has been seen to have a better trend than TAVR, even in intermediate-risk patients. In fact, Miceli et al. demonstrated a lower rate of mortality and stroke in patients undergoing sutureless aortic valve replacement surgery as well as a better hemodynamic performance linked to the lower rate of perivalvular leaks (5.4% vs. 81.1%, *p*-value < 0.001) [54]. Referring to the most recent data present in the literature, we analyzed the results of the two procedures (SAVR with sutureless and TAVR) by comparing echocardiographic data, such as peak and mean transvalvular gradients, presence of perivalvular leaks, and pacemaker implantation, and clinical data, such as 30-day mortality, onset of stroke, and acute kidney injury.

### 4.1. Peak and Mean Transvalvular Gradients

Munuretto et al., in their multicenter study on elderly patients at intermediate risk, demonstrated how patients undergoing sutureless surgery had mean and peak transvalvular gradients of 12.2 ± 5.7 and 22.3 ± 9, respectively, at 60 months of follow-up, while patients undergoing TAVR had mean and peak transvalvular gradients of 13.27 ± 6.3 and 23.51 ± 9.5, respectively. Furthermore, a more statistically significant finding was a severe (≤0.65 cm^2^/m^2^) patient–prosthesis mismatch (PPM) observed in 12 (4.1) patients undergoing sutureless surgery and in 19 (6.5%) patients undergoing TAVR [55]. Furthermore, this result overturns what was previously demonstrated by the same author in a previous multicenter study in which the average and maximum transvalvular gradients in patients undergoing TAVR were lower than those in patients undergoing sutureless surgery, although the analysis was conducted in the peri- and immediate post-operative period [56]. Similarly, other authors, in comparing the hemodynamic performance of sutureless and TAVR, had highlighted a lower mean and maximum transvalvular gradient in patients undergoing a percutaneous procedure [57], as well as a lower number of PPMs [58]. However, even in this case, the data were related to the patient’s discharge and not related to further follow-up. Therefore, we believe that attention should be paid to the long-term (>24 months) evaluation of maximum and mean transvalvular gradients.

### 4.2. Perivalvular Leaks

The removal of the aortic cusps, the decalcification of the annulus, and the subsequent implantation of the prosthesis guarantee greater adherence of sutureless aortic prosthesis to the aortic root. For this reason, the presence of perivalvular leaks is always greater in the TAVR group compared to the group of patients undergoing sutureless, as demonstrated by numerous recent meta-analyses [59,60,61]. Having established that the presence of severe perivalvular leaks that generate aortic insufficiency with grade > 2 constitutes the strongest independent predictor of 1-year mortality [62], attention was placed on the development of techniques that can reduce the degree of aortic insufficiency post-TAVR. Landes et al. conducted a retrospective study on the techniques used in patients with aortic insufficiency with grade > 2, which involved the use of redo-TAVR, plug, or balloon valvuloplasty, demonstrating that the degree of reduction in aortic insufficiency and mortality were more favorable in redo-TAVR than with the other two methods. However, redo-TAVR can increase the risk of patient–prosthesis mismatch, coronary obstruction, or access difficulties [63]. Although technological progress will lead to greater refinement of the techniques as well as greater adhesion of the percutaneous prostheses to the native aortic valve, the results in terms of perivalvular leaks of TAVR are currently lower than those of sutureless ones.

### 4.3. Pacemaker Implantation

While the results relating to perivalvular leaks appear quite clear between the two groups, the same cannot be said regarding the incidence of pacemaker implantation. A meta-analysis of six comparative matched studies using propensity score matching (identified 1462 patients in that 731 patients underwent sutureless and 731 patients underwent a TAVR) conducted by Meco and others revealed no differences in pacemaker implantation between the two groups (OR 1.06, 95% CI 0.54–2.08; *p* = 0.86) [64]. More recently, Zubarevich et al. analyzed the results of 248 consecutive patients who underwent either sutureless or TAVR at our institution between April 2018 and June 2021, and although the TAVR group had a numerically larger sample (169 vs. 79), no differences were highlighted between the two groups [65]. On the contrary, Munuretto et al., in their recent meta-analysis conducted on patients with isolated aortic stenosis at intermediate risk, demonstrated a statistically higher rate of pacemaker implantation in patients undergoing TAVR [55]. However, none of the recent manuscripts on this topic associate sutureless prostheses with a greater risk of pacemaker implantation compared to TAVR. Furthermore, the variability relating to the TAVR group seems to be explained by the technique and choice of device used. In fact, Edwards Sapiens prostheses have been associated with a lower incidence of pacemaker implantation, as well as not performing balloon-dilation of the aortic valve before balloon-expansion of the prosthesis or positioning it slightly towards the coronary artery, which may extrude the calcified native cusps less toward the region of least resistance, causing structural damage and edema within the conduction system [66].

The retrospective studies (excluding meta-analyses and systematic reviews) cited are summarized in Table 1.

### 4.4. Clinical Outcomes

A recent meta-analysis performed by Barili et al. [67], including seven trials, showed that TAVR had a lower incidence of the composite endpoint of death or stroke in the first 6 months, with an HR reversal after 24 months favoring SAVR. This was confirmed for all-cause death. TAVR was also associated with an increased incidence of rehospitalization after 6 months, which worsened after 24 months. Although it could appear that there is no difference between TAVR and SAVR in the 5-year cumulative results, TAVR shows a strong protective effect in the short term that runs out after 1 year. TAVR becomes a risk factor for all-cause mortality and the composite endpoint after 24 months and for rehospitalization after 6 months. Surgical aortic valve replacement (SAVR) has an intrinsic increased risk of complications in the first months, related, for example, to extracorporeal circulation and surgical incisions, risks that decrease soon after surgery. This result is in contrast with those of single randomized controlled trials (RCTs) and other published metanalyses that use summary data [68]. The relatively short follow-up time in RCTs on intermediate- and low-risk groups limited results between 2 and 5 years because only the two trials on high-risk and one small trial on low-risk reached the 5-year follow-up [23,24,25,26,27,28,29,30,31]. The PARTNER 2A trials and the PARTNER 3 increased the 5-year follow-up population from 1776 patients to 3808 [29,34]. The PARTNER 2 A trial enrolled 2032 intermediate-risk patients with severe, symptomatic aortic stenosis at 57 centers. Patients were stratified according to intended transfemoral or transthoracic access (76.3% and 23.7%, respectively) and were randomly assigned to undergo either TAVR or surgical replacement. Among patients with aortic stenosis who were at intermediate surgical risk, there was no significant difference in the incidence of death or disabling stroke at 5 years after TAVR as compared with surgical aortic-valve replacement. The PARTNER 3 trial randomly assigned 1000 patients (1:1) to transfemoral TAVR with the SAPIEN 3 valve versus surgery (mean Society of Thoracic Surgeons score: 1.9%; mean age: 73 years) with clinical and echocardiography follow-up at 30 days and at 1 and 2 years. This trial concluded that at 2 years, the primary endpoint remained significantly lower with TAVR versus surgery, but initial differences in death and stroke favoring TAVR were diminished, and patients who underwent TAVR had increased valve thrombosis.

Partially overlapping results emerged in retrospective and meta-analysis studies between patients undergoing TAVR and those undergoing sutureless. In particular, it emerges that if 30-day mortality is overlapping in the two groups, the risk of mortality in the follow-up is higher in the group undergoing TAVR, partly explained by the greater age of patients who undergo percutaneous treatment [51,69] but probably linked to the high rate of PVL and PMK implantation in the TAVR group which certainly affects hemodynamic compliance. These results are also confirmed in intermediate-risk patients, confirming the need to evaluate the appropriate treatment for the individual patient, also based on the experience of the center [69]. However, the treatment of intermediate-risk patients remains a field of scientific interest that needs to be expanded and strengthened since the amount of data available to create a standardized algorithm is still limited.

### 4.5. Minimally Invasive AVR versus TAVI

A lot of studies assessed the benefit of minimally invasive surgery AVR when compared with conventional AVR. lmeida et al. [70], in an excellent meta-analysis and meta-regression on 1303 patients, showed that minimally invasive aortic replacement is a valid alternative to conventional AVR, reducing hospital stay and incidence of hemorrhagic events. Despite significantly greater aortic cross-clamp and cardiopulmonary bypass time in patients who underwent minimally invasive surgery, this is not associated with an increase in adverse clinical effects. Accordingly, Angelini et al. [71], in an interesting multicenter, international, randomized controlled trial titled COMICS including patients aged ≥18 and <85 years undergoing elective or urgent isolated coronary artery bypass grafting (CABG), isolated aortic valve replacement (AVR) surgery, or CABG + AVR surgery, showed that minimally invasive surgery reduces the relative risk of primary outcome events by about 25%. They defined as primary outcomes events the following 12 post-operative serious adverse events: death, myocardial infarction (MI; suspected events were documented by serum troponin concentrations and electrocardiograph recording (ECG), but the latter was not provided), stroke (report of brain imaging by CT or MRI, in association with new-onset focal or generalized neurological deficit), gut infarction (diagnosed by laparotomy or post mortem), stage 3 AKI or need for haemofiltration, reintubation, tracheostomy, mechanical ventilation >48 h, reoperation, percutaneous intervention, sternal wound infection with dehiscence, and septicemia confirmed by microbiology. The right anterior thoracotomy (RAT) showed excellent results in terms of mortality and rate of post-operative complications when compared with different surgical techniques [53]. The association between RAT and sutureless appears to be better even than TAVR, although there are still few comparison studies available. Doyle et al. developed a propensity-matched meta-analysis study in which they demonstrated that with the same surgical risk, patients undergoing minimally invasive surgical treatment had a lower rate of pacemaker implantation (OR 0.2; 95% CI 0.09–0.51), aortic insufficiency linked to the presence of periprosthetic leaks (OR 0.05; 95% CI 0.01–0.19), while a higher rate of acute renal failure was highlighted in patients undergoing minimally invasive surgical treatment with a consequent negative impact on the length of hospital stay (OR 3.15; 95% CI 2.07–4.80). However, the mortality rate between the two groups was comparable (OR 0.90; 95% CI 0.35–2.26) [72]. Therefore, what emerges is a substantial difference in terms of better clinical and hemodynamic outcomes in patients undergoing minimally invasive surgery compared to traditional surgery and completely comparable, and in some respects superior, to those of patients undergoing TAVR [53]. Even more recently, a retrospective Korean study has demonstrated better results of minimally invasive surgery (particularly in the right minithoracotomy approach) in elderly patients at high surgical risk by comparing the length of hospital stay, in-hospital mortality, all-cause mortality, and other major post-operative complications [73]. It is hoped, therefore, that comparison studies between the two groups can be increasingly numerous so as to be able to define with ever greater accuracy the therapeutic treatment suitable for the clinical and anatomical characteristics of the patients.

### 4.6. Structural Durability of TAVAR versus SAVAR

Both SAVR and TAVR use bioprosthetic valves, with the SAVR valve being a fixed stent with an estimated life span of 15 years and the TAVR valve being capable of expanding and collapsing [74]. However, TAVR being the newer procedure, with the first valve implanted in 2002 by Alan Criber and developments in the technique and valves having spanned only just under 20 years, the life span of the TAVR valve is still uncertain. Most studies that compare TAVR to SAVR valves report data of only up to 5 or 6 years, making an assessment of valve durability beyond that time frame difficult to determine. In an interesting study, Ler et al. showed that TAVR valves appear to be more susceptible to structural valve deterioration and thus potentially less structurally durable than SAVR valves; therefore, they may be associated with higher rates of moderate or severe aortic regurgitation, paravalvular regurgitation, and reintervention in the 1-year, 2–3-year, and 5-year periods [75]. However, it is not possible to compare the structural durability between TAVAR and SuAVR because there are very few data on the durability of SuAVR valves. Considering their substantial recent introduction to the market (approved by the FDA in 2016), the results on the durability of sutureless prostheses are still lacking. One of the studies with the longest follow-up (10 years) analyzed the results of sutureless prosthesis implantation in 547 patients at low surgical risk and showed a structural deterioration of the valve only in 23 patients, of whom 19 required re-intervention, with an average freedom from re-intervention of 10.3 years [76]. Szece and colleagues performed an 11-year follow-up in 468 patients undergoing sutureless prosthesis implantation with intermediate surgical risk (Euroscore II 5.1 ± 5.5) and did not highlight any structural valve degeneration [77]. Furthermore, Werner and colleagues, in a comparative analysis of 1070 patients (Trifecta *n* = 298, Intuity *n* = 772) followed up for 4 years, highlighted a higher risk of structural valve degeneration in patients undergoing Trifecta implantation compared to the Intuity prosthesis (Trifecta *n* = 23, Intuity *n* = 4) and cumulative incidence of re-interventions at 1 and 5 years was 1.85 (95% CI: 0.7–4.05) and 3.87 [1.87–6.99] in the Trifecta group and 2.30 [1.37–3.62] and 3.02 [1.83–4.67] in the Intuity group. At 7 and 8 years, it increased to 22.26 [12.86–33.27] and 37.28 [20.26–54.33] in the Trifecta group and to 5.65 [2.98–9.53] in the Intuity group [78]. Therefore, the data currently available suggest that the durability of the sutureless prosthesis is closer to that of the Perimount prosthesis, a biological prosthesis with currently the longest follow-up present in the literature with a freedom from SVD of 94.2% at 10 years, 78.6% at 15 years, and 48.5% at 20 years (in the work of Pollari and colleagues, a 5-year freedom from SVD of 98.5% emerged in the sutureless prosthesis) [76].

Finally, SuAVR, as well as TAVI valves, are tall with frames that may extend into the ascending aorta. This can be problematic for future surgery or in valve-in-valve replacement when they inevitably fail.

## 5. Conclusions

In intermediate-risk patients with severe aortic stenosis, minimally invasive aortic valve replacement with sutureless prosthesis could be a better treatment, particularly in patients with morphological predictors of paravalvular leak after TAVR (asymmetrical distribution of calcium and/or massive and bulky annular calcification). However, this hypothesis needs to be confirmed by RCTs comparing SuAVR with TAVI.

## 6. Limitations

There are no randomized controlled studies in this somewhat limited group of patients; furthermore, there are relatively few data on SuAVR. The biggest limitation is that any comparisons of SuAVR versus TAVI are based on observational data and the use of statistical techniques such as propensity score matching. This, however, cannot fully account for heterogeneity, particularly of factors that were not measured, and there likely remains selection bias for patients who underwent TAVI or surgery.

Comparing implantation techniques as a class is, therefore, challenging. In addition, it is important to recognize that the technique by which a valve is implanted is only one factor, and different surgical techniques need to be considered. A Perceval valve may perform very differently than an Intuity valve in the same way that different surgical valves and TAVI valves are known to have different characteristics and risks associated with implantation.

## Figures and Tables

**Table 1:** The retrospective studies (excluding meta-analyses and systematic reviews) cited in the review.

Study/Year	Study Period	Type of Study	Study Size	Risk Class	Peak and Mean Transvalvular Gradients (Mean ± SD) mmHg	Perivalvular Leaks	Pace-Maker Implantation
Muneretto C. et al., 2020 [54]	2008–2015	retrospective study	Sutureless: 481 patients TAVR: 486 patients	intermediate risk	Peak Sutureless: 23.2 ± 9.3 Mean Sutureless: 11.1 ± 5.7 Peak TAVR: 22.9 ± 8.4 Mean TAVR: 10.6 ± 4.9	Grade > II Sutureless: 5 patients TAVR: 36 patients	Sutureless: 29 patients TAVR: 60 patients
Muneretto C. et al., 2015 [55]	2007–2014	observational, retrospective multicenter cohort study	Postmatching Sutureless: 204 patients TAVR: 204 patients	Intermediate to high risk	Peak Sutureless: 19.52 ± 12.45 Mean Sutureless: 10.8 ± 6.8 Peak TAVR: 14.34 ± 7.5 Mean TAVR: 7.6 ± 4.2	Grade > II Sutureless: 4 patients TAVR: 18 patients	Sutureless: 20 patients TAVR: 30 patients
Vilalta V. et al., 2021 [56]	2011–2020	retrospective study	Postmatching Sutureless: 171 patients TAVR: 171 patients	low risk	Mean Sutureless: 49.4 ± 16.4 Mean TAVR: 46.0 ± 17.2	Moderate-severe Sutureless: 1/159 patients TAVR: 1/94 patients	Sutureless: 23 patients TAVR: 34 patients
Kamperidis V. et al, 2015 [57]	2007–2013	retrospective study	Postmatching Sutureless: 40 patients TAVR: 40 patients	High risk	Mean Sutureless: 10.72 ± 4.01 Mean TAVR: 8.14 ± 4.21	Grade > II Sutureless: 2 patients TAVR: 9 patients	Sutureless: 1 patient TAVR: 3 patients

## Data Availability

All articles cited in this review are available on PubMed.
